# Multicore, SDS-Based Polyelectrolyte Nanocapsules as Novel Nanocarriers for Paclitaxel to Reduce Cardiotoxicity by Protecting the Mitochondria

**DOI:** 10.3390/ijms26030901

**Published:** 2025-01-22

**Authors:** Marzena Szwed, Anastazja Poczta-Krawczyk, Katarzyna D. Kania, Kacper Wiktorowski, Kamila Podsiadło, Agnieszka Marczak, Krzysztof Szczepanowicz

**Affiliations:** 1 Department of Medical Biophysics, Institute of Biophysics, Faculty of Biology and Environmental Protection, University of Lodz, Pomorska 141/143 St, 90-236 Lodz, Poland; 2 Laboratory of Virology, Institute for Medical Biology, Polish Academy of Sciences, Lodowa 106A St, 93-232 Lodz, Poland; 3 Department of Diagnostic Techniques in Pathomorphology, Medical University of Lodz, Pomorska 251, 92-213 Lodz, Poland; 4 Student’s Scientific Circle of Young Biophysicists, Institute of Biophysics, Faculty of Biology and Environmental Protection, University of Lodz, Pomorska 141/143 St, 90-236 Lodz, Poland; 5 Jerzy Haber Institute of Catalysis and Surface Chemistry, Polish Academy of Sciences, Niezapominajek 8 St, 30-239 Kraków, Poland

**Keywords:** nanoparticles, LbL technique, cardiovascular damage, taxanes

## Abstract

The clinical application of paclitaxel (PTX), a widely used anticancer drug, is constrained by cardiac arrhythmias and disruptions in vascular homeostasis. To mitigate the non-specific, high toxicity of PTX towards cardiomyocytes, we propose the application of newly synthesized SDS-based polyelectrolyte multicore nanocapsules. This study aims to verify the hypothesis that SDS-based NCs can mitigate the cytotoxic effects of PTX on cardiac cells and serve as effective nanocarriers for this drug. We investigated two types of multicore NCs with differing polyelectrolyte coatings: poly-L-lysine (PLL) and a combination of PLL with poly-L-glutamic acid (PGA). The cytotoxicity of the formulated nanosystems was evaluated using HL-1 cardiomyocytes. Oxygraphy, flow cytometry, spectrophotometry, spectrofluorimetry, fluorescence microscopy, and RT-PCR were employed to assess disruptions in cardiac cellular homeostasis. Our data revealed that, among the tested NCs, SDS/PLL/PGA/PTX exhibited reduced cardiotoxicity and were better tolerated by HL−1 cardiomyocytes compared to SDS/PLL/PTX or PTX alone. In addition, SDS/PLL/PGA/PTX showed a marginal disruption of mitochondria’s homeostasis, and no changes in APT level and intracellular calcium concentrations were observed. These findings underscore the potential of SDS-based multicore nanocarriers in anticancer therapy, particularly due to diminished cardiotoxicity and long-term stability in the biological fluids.

## 1. Introduction

In recent years, advances in systemic antineoplastic drug therapies and the introduction of neoadjuvant strategies into clinical practice have significantly improved the survival rates for cancer patients [1,2]. However, these improvements do not necessarily translate into an enhanced quality of life for individuals diagnosed with various malignancies, and many patients experience severe side effects associated with cancer treatments. Among the complications, hypersensitivity, neutropenia, and myelosuppression are prevalent, but cardiotoxicity remains one of the most common and concerning issues developing during chemotherapy or radiotherapy [3]. The most frequently observed cardiac disorders include ischemia, venous thromboembolism, hypertension, and arrhythmia [4]. Furthermore, cardiovascular risks, such as myocardial dysfunction and thromboembolism, can lead to systemic and pulmonary hypertension, as well as accelerated coronary artery disease. Among the highly effective chemotherapeutic agents, paclitaxel (PTX) is a tubulin-binding anticancer drug that plays a pivotal role in inhibiting cancer cell mitosis [5]. However, its use is associated with adverse effects, including structural and functional alterations in the myofibrils of cardiomyocytes [6]. Currently, PTX is widely employed in the treatment of various aggressive and metastatic solid tumors, such as breast, ovarian, bladder, and prostate cancer [7,8]. The risk of cardiovascular disease during the PTX treatment can be qualified by two primary factors. Firstly, PTX is poorly soluble in water, and the use of conventional solvents like polyoxyethylene castor oil during its administration is necessary [9,10]. This process can lead to the release of histamine, which results in cardiac conduction system disorders and arrhythmias. Secondly, PTX is frequently combined with other cytotoxic drugs, such as anthracyclines, significantly increasing the risk of heart failure due to the generation of free radicals by these anticancer agents [11,12]. Although the cardiotoxic effects of PTX are often reversible, the precise mechanisms underlying these effects remain unclear. It has been shown that chemotherapeutic drugs with antimitotic properties would, in parallel, disrupt mitochondrial homeostasis. For example, during PTX’s mode of action, cells arrested in the M phase exhibit compromised mitochondrial function. This dysfunction is characterized by reduced mitochondrial membrane potential (Δψm), increased SUMOylation and acetylation of mitochondrial proteins, and a heightened metabolic dependence on glycolysis [13].

Nanotechnology-based drug delivery systems have recently been proposed as a strategy to ease the severe side effects associated with PTX administration. Nanoparticles (NPs) have been primarily designed first to increase the short circulation time of the drug in the bloodstream [14,15] and, second, to enhance the targeted delivery of chemotherapeutic agents directly to cancer cells [16]. Additionally, NPs-dependent therapies can encapsulate drugs within nanocarriers, allowing for the precise targeting of cancer cells and reducing off-target effects [17]. For instance, Abraxane, an albumin-bound, solvent-free PTX NP, was approved by the Food and Drug Administration (FDA) in 2005 for breast cancer therapy. Abraxane demonstrated a significantly higher, maximum tolerated dose compared to the commercial formulation of PTX, reducing several Taxol-associated adverse effects, such as hypotension and bradycardia [18,19]. Another example is Genexol-PM, a lyophilized polymeric micellar nanosystem containing PTX, which was launched in 2007 in the Korean market for breast cancer treatment. However, this formulation has been linked to a high accumulation of the drug in cardiac tissue, significantly increasing the risk of developing congestive cardiovascular disease [20].

A convenient method for the fabrication of biodegradable polymeric nanocarriers is the sequential adsorption of oppositely charged polyelectrolytes, known as the layer-by-layer (LbL) technique [21,22]. The use of polyelectrolyte multilayer nanocapsules with liquid cores is supported by their ability to regulate chemical modifications and multifunctionality. So far, the surfactants used during the synthesis of polyelectrolyte nanocapsule cores, such as docusate sodium salt (AOT) or dioctadecyl dimethylammonium bromide (DODBR), have been mostly oil soluble [23]. A significant obstacle to employing these surfactants during LbL nanocapsule (NC) synthesis lies in the necessity of using highly toxic organic solvents, such as chloroform, cyclohexane, or ethylene glycol. Since the biomedical application of nanoparticles demands the complete elimination of toxic compounds, the use of oil-soluble surfactants during LbL nanocapsule synthesis becomes highly questionable. Considering the intended application of the drug nanocarrier targeted to cancer cells, we had proposed an alternative and safer approach involving the use of water-soluble surfactants, for instance SDS [24]. This surfactant forms bulk complexes (micelles/polyelectrolyte) with oppositely charged polyelectrolytes. In addition, such complexes can also serve as the core of multicore polyelectrolyte nanocapsules, with the micellar interior providing a perfect environment for hydrophobic drugs.

In our previous study [24], we demonstrated that NCs prepared using the LbL procedure, based on the water-soluble surfactant sodium dodecyl sulphate (SDS), can effectively release PTX and selectively kill cancer cells. PTX delivered as SDS/PLL/PTX or SDS/PLL/PGA/PTX exhibited cytotoxic properties, suppressing the proliferation of breast cancer cells through the induction of programmed cell death. In parallel, experiments performed on the human microvascular endothelial cell line (HMEC-1) revealed a higher resistance to these forms of PTX compared to tumor cells.

The present study is the first to employ a comprehensive set of functional assays to evaluate the effects of PTX encapsulated in SDS-based polyelectrolyte multicore nanocarriers on cardiac myocytes. Specifically, our objectives were as follows: (I) to investigate the impact of SDS/PLL/PTX and SDS/PLL/PGA/PTX on cardiomyocyte homeostasis; and (II) to determine how mitochondrial bioenergetics is altered by the cytotoxic effects of PTX released from these nanosystems.

Our recent observations showed that HMEC-1 cells, representing non-cancerous endothelial cells, did not respond as significantly to the SDS/PLL/PTX or SDS/PLL/PGA/PTX treatments as triple-negative breast cancer cells responded. This suggests that SDS-based polyelectrolyte NCs minimally disrupt endothelial function, offering a potential new strategy for PTX delivery in clinical settings. In light of the recent literature, we examined here not only the markers of stress responses triggered by PTX in cardiomyocytes, including calcium release, ATP content, DNA synthesis, and alterations in the expression of cardio sensitivity-related genes, but mitochondrial damage induced by PTX released from SDS/PLL/PTX or SDS/PLL/PGA/PTX NCs.

Based on our prior findings regarding the concentration-dependent effects of SDS-based nanocarriers on human breast cancer cells, we examined the IC_50_ concentrations of the free drug and PTX encapsulated in the investigated nanosystems. We identified that the cytotoxic effect of PTX was dependent on the form of the nanocarriers used for drug encapsulation and the period of exposure to chemotherapeutic agent. To enhance our understanding of the observed differences between the various forms of PTX, we conducted a detailed oxygraphy-based analysis of mitochondrial homeostasis. Notably, we identified several changes in oxygen consumption rate and Δψm that are likely relevant to the cytotoxic effects of these substances on cardiac myocytes. These alterations agreed with the mRNA expression of genes involved in mitochondrial-dependent cell death pathways as well as cardiac tissue homeostasis.

## 2. Results

### 2.1. Physiochemical Properties of the SDS-Based NCs

**Figure 1:**
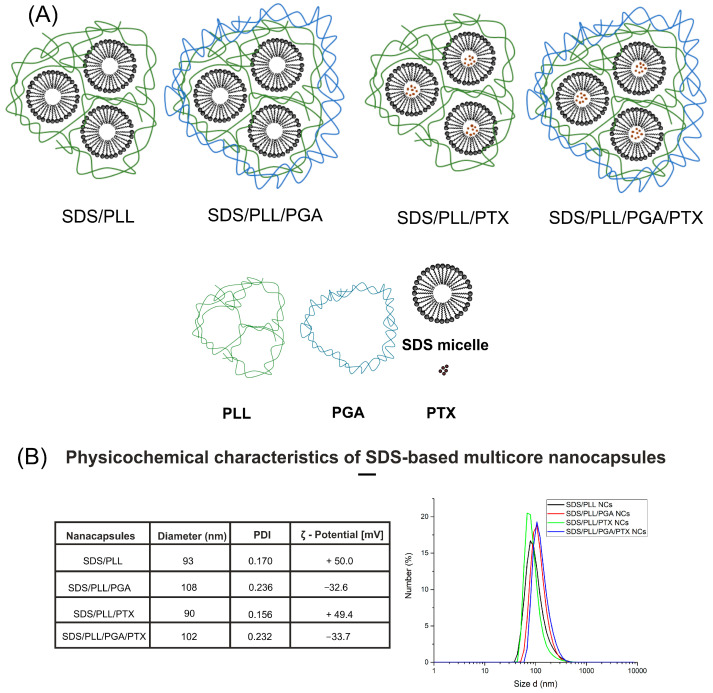
Schematic illustration of SDS-based nanocarriers and their physicochemical properties. (**A**) Structural representation of empty and PTX-loaded SDS-based NCs along with the key components of the nanocarriers. (**B**) Representative data for diameter, polydispersity index (PDI), and zeta potential of the NCs together with the exemplary diameter size distribution histograms.

The hydrodynamic diameter of SDS-based NCs, both with and without paclitaxel (PTX), ranged from 90 to 108 nm, exhibiting a narrow size distribution (PDI < 0.05). SDS/PLL NCs, with or without encapsulated drug, were positively charged, with a zeta potential of +49.4 to +50 mV (Figure 1A,B). By contrast, SDS/PLL/PGA NCs with encapsulated PTX or empty ones exhibited a negative zeta potential of −33.7 and −32.6 mV, respectively. The NC particles remained stable in sodium chloride solutions when stored at room temperature for over 24 h and no alterations were observed in either the size distribution or the zeta potential analysis of PTX encapsulation efficiency in the NCs was determined to be 100%.

### 2.2. PTX Trapped in SDS-Based Polyelectrolyte NCs Showed Diminished Cytotoxicity Towards HL-1 Cardiomyocytes in Comparison to Free Drug

**Figure 2:**
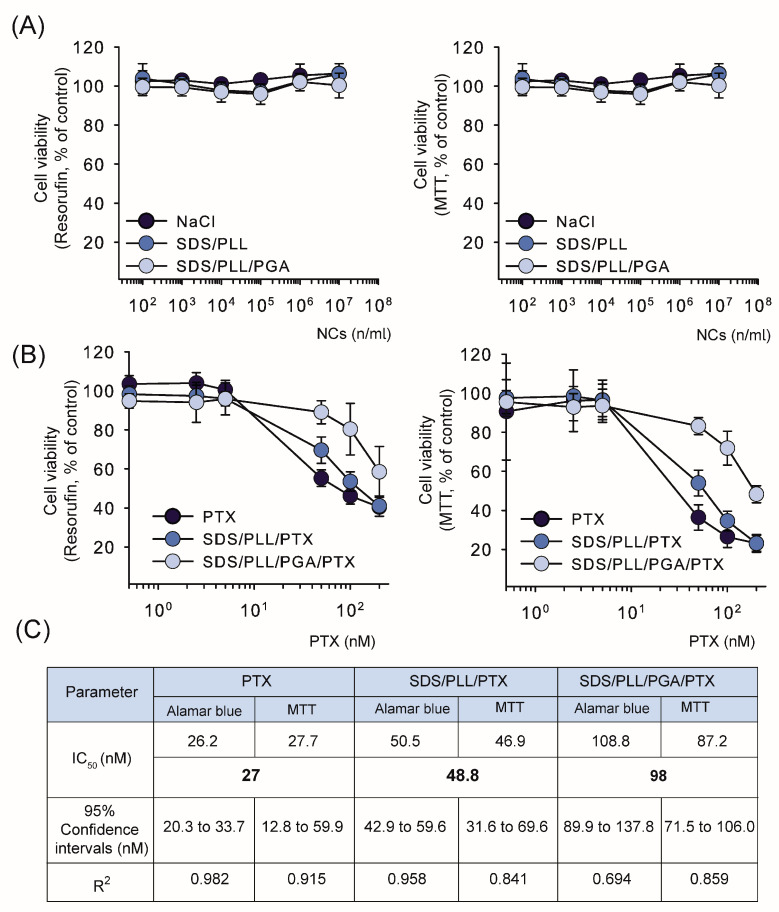
Cytotoxic effects of SDS/PLL and SDS/PLL/PGA nanocapsules (with and without PTX) on HL-1 cardiac cells. (**A**) Cell viability of HL-1 cardiomyocytes after exposure to empty SDS-based NCs for up to 72 h, assessed using the Alamar Blue assay (**left** panel) and the MTT test (**right** panel). (**B**) Dose-dependent cytotoxicity of SDS/PLL/PTX, SDS/PLL/PGA/PTX, and free PTX following 72 h of treatment, evaluated using the Alamar Blue assay (**left** panel) and the MTT test (**right** panel). Results are expressed as percentages relative to untreated control cells and are presented as mean values ± SD from four independent experiments. (**C**) IC_50_ values for free PTX, SDS/PLL/PTX, and SDS/PLL/PGA/PTX in HL-1 cells, calculated from cell viability curves.

The cytotoxicity of NCs loaded with PTX and free drug against HL-1 cardiomyocytes was assessed using Alamar Blue (resazurin converted into fluorescent resorufin) and MTT assays following treatments for up to 72 h. Cytotoxic effects of empty NCs (SDS/PLL and SDS/PLL/PGA) were first evaluated and both formulations were non-toxic at tested concentrations (Figure 2A). PTX-loaded NCs exhibited different cytotoxic profiles compared to free PTX depending on the assay used (Figure 2B). For example, the MTT assay revealed a 20% reduction in cell viability with 200 nM free PTX treatment. On the other hand, both performed cytotoxicity assays allowed us to estimate that the respective SDS/PLL/PTX and SDS/PLL/PGA/PTX IC_50_ values of HL1 cells were 48.8 nM and 98.1 nM, compared to 26.5 nM for free PTX. This means that the drug encapsulated in SDS/PLL/PGA nanocarrier revealed a diminished cytotoxicity (*p* < 0.001), with IC_50_ values approximately twofold and fourfold higher than SDS/PLL/PTX and free PTX, respectively (Figure 2C).

### 2.3. Morphology of HL-1 Cells Treated with Investigated Forms of PTX

**Figure 3:**
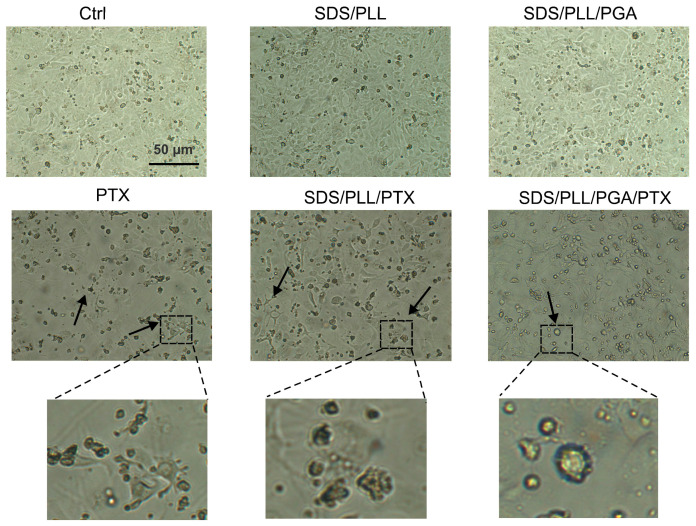
Morphological changes in HL-1 cells induced by PTX and SDS-based NCs. Inverted phase-contrast microscopy images of HL-1 cells treated for 48 h with IC_50_ concentrations of SDS/PLL, SDS/PLL/PGA, free PTX, SDS/PLL/PTX, or SDS/PLL/PGA/PTX. The scale bar represents 50 µm. Black arrows indicate clumped and shrunken cells. Magnification: 100×. The photos were acquired using inverted microscope equipped with digital camera (Olympus IX70, Tokyo, Japan).

The morphology of HL-1 cardiomyocytes treated with free PTX, SDS/PLL/PTX, SDS/PLL/PGA/PTX, or empty NCs (SDS/PLL and SDS/PLL/PGA) was assessed via light microscopy (Figure 3). Cells exposed to empty NCs displayed no morphological changes. However, exposure to free PTX or SDS/PLL/PTX for up to 48 h resulted in decreased cell density. Notably, free PTX exhibited the most pronounced toxic effects, including a severe reduction in cardiomyocyte proliferation, membrane protrusion, and extension. By contrast, SDS/PLL/PTX caused mild detachment and cell shrinkage, while SDS/PLL/PGA/PTX showed no significant reduction in cell density at this time point.

### 2.4. The Rate of ATP and DNA Synthesis in Cardiomyocyte Cell Cultures Was Reduced by Investigated Compounds

**Figure 4:**
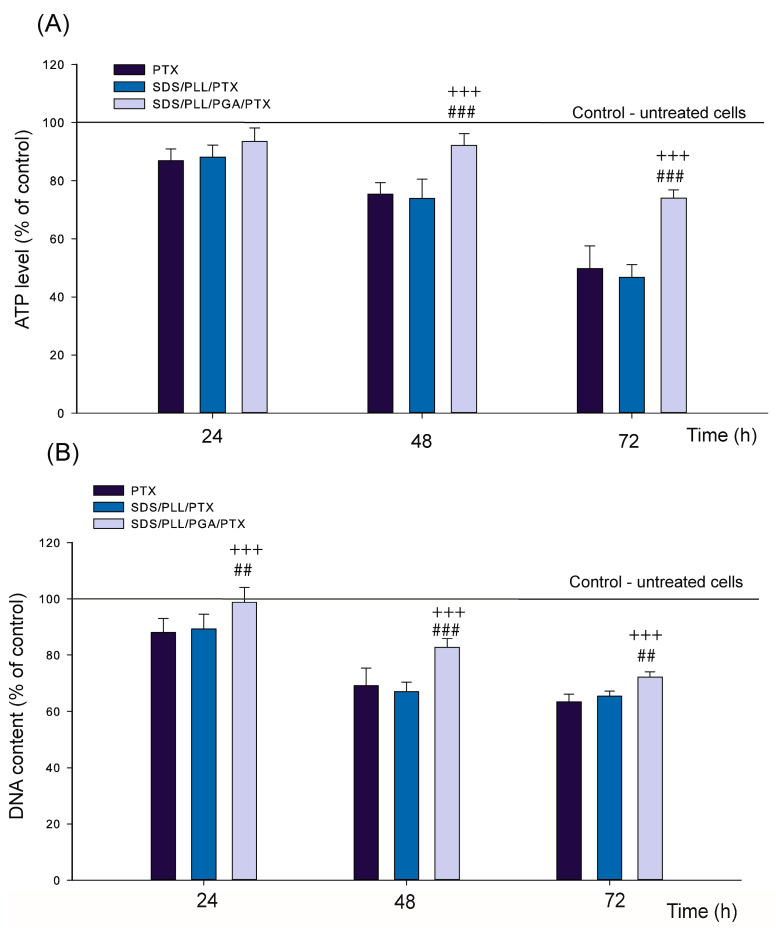
DNA and ATP synthesis alterations in HL-1 cells treated with free PTX or PTX encapsulated in SDS-based NCs. Data are presented as a mean ± SD from four independent experiments. All values are normalized to untreated control cells (set at 100%). +++ *p* < 0.001 indicates statistically significant differences between treatments PTX or SDS/PLL/PTX, SDS/PLL/PGA/PTX-treated cells, and ## *p* < 0.01, ### *p* < 0.001 denotes significant differences between SDS/PLL/PTX and SDS/PLL/PGA/PTX treatments (**A**) Cellular ATP levels in HL-1 cardiomyocytes treated with IC_50_ concentrations of PTX alone, SDS/PLL/PTX, or SDS/PLL/PGA/PTX for 24, 48, and 72 h. (**B**) DNA content in HL-1 cell cultures after treatment with SDS/PLL/PTX, SDS/PLL/PGA/PTX, or free PTX (IC_50_ concentrations) for 24, 48, and 72 h. DNA levels were quantified via Hoechst 33258 staining of permeabilized cells, with fluorescence measured at excitation 355 nm and emission 460 nm. Fluorescence from untreated control cells was set at 100%.

Changes in cellular homeostasis were evaluated by measuring intracellular ATP levels [25] and DNA content [26], as disturbances in these parameters serve as early indicators of cytotoxicity. Treatment with SDS/PLL/PTX and SDS/PLL/PGA/PTX at IC_50_ concentrations demonstrated time-dependent reductions in ATP levels, as assessed by the CellTiter-Glo^®^ assay (Figure 4A). Both free PTX and SDS/PLL/PTX decreased ATP content by 46% after 72 h of incubation. Interestingly, SDS/PLL/PGA/PTX exhibited significantly lower ATP reduction compared to free PTX at 48 and 72 h (*p* < 0.01), with no measurable changes observed for any compound at 24 h. This is slightly opposite in the analysis of the DNA content measured with Hoechst 33258 (Figure 4B). Notably, after 24 h of incubation, SDS/PLL/PGA/PTX displayed statistically significant differences compared to free PTX (*p* < 0.001). The most substantial reduction in DNA content (63%) was observed following 72 h of exposure to free PTX (Figure 4B).

### 2.5. The Alteration of Intracellular Ca^2+^ Concentration in Free PTX and PTX Encapsulated in SDS-Based NCs

To evaluate whether cytosolic calcium ion (Ca^2+^) dysregulation contributed to cellular homeostasis disturbances triggered by SDS/PLL/PTX, SDS/PLL/PGA/PTX, or free PTX (applied at IC_50_ concentrations), the level of free Ca^2+^ was assessed using Fluo-4 Direct™ staining. Thapsigargin [27], a known SERCA pump inhibitor that elevates intracellular Ca^2+^ by blocking its transport into the endoplasmic reticulum [28], was used as a positive control. Upon treatment with thapsigargin for 24 h, a 2.7-fold increase in Fluo-4 Direct™ fluorescence intensity was observed.

**Figure 5:**
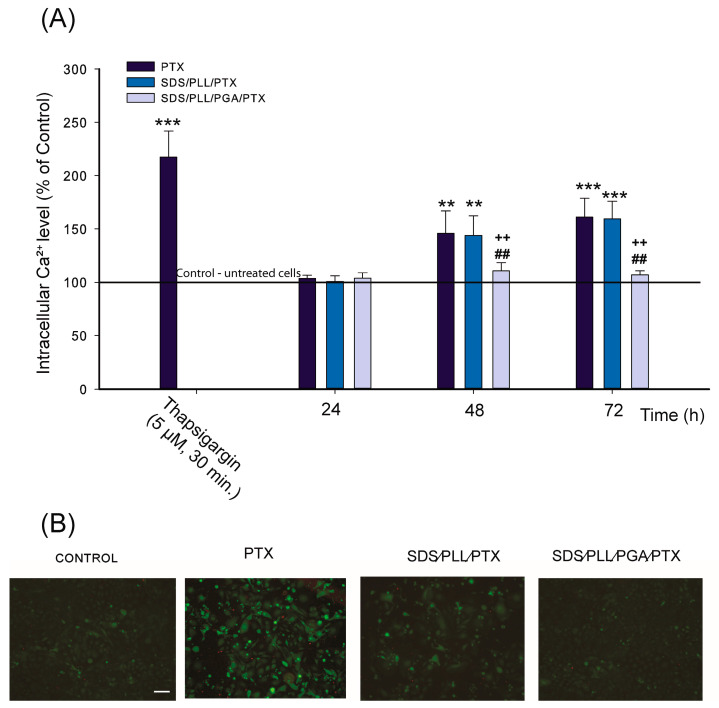
Effects of PTX and the drug trapped in SDS-based NCs on intracellular Ca^2+^ levels in HL-1 cells. (**A**) Intensity of Fluo-4 Direct™ fluorescence was measured to evaluate cytosolic Ca^2+^ levels. Fluorescence intensity in control cells was set at 100%. Data represent the mean ± SD of four independent experiments. *** *p* < 0.001, ** *p* < 0.01 indicates significant differences compared to control cells; ++ *p* < 0.01 denotes differences between treatments PTX or SDS/PLL/PTX, SDS/PLL/PGA/PTX-treated cells; ## *p* < 0.01 highlights differences between the SDS/PLL/PTX and SDS/PLL/PGA/PTX treatments. (**B**) Fluorescence microscopy images of HL-1 cells stained with Fluo-4 Direct™ probe after 24 h of treatment with PTX, SDS/PLL/PTX, or SDS/PLL/PGA/PTX. Images were captured using an Olympus IX70 inverted fluorescence microscope (Olympus, Tokyo, Japan) at 400× magnification. The scale bar represents 50 µm.

Among the tested compounds, SDS/PLL/PTX and free PTX induced the most substantial increase in cytoplasmic Ca^2+^ levels, with ~45% and ~60% increases detected after 48 and 72 h of incubation, respectively. As shown in Figure 5A, no significant changes in Ca^2+^ levels were observed in cells treated with SDS/PLL/PGA/PTX across 24, 48, and 72 h. These data are in agreement with the microscopic observations performed for cardiomyocytes exposed to examined compounds and stained with Fluo-4 Direct™. Figure 5B shows the comparable, fluorescence signal recorded for Hl-1 cells treated with SDS/PLL/PTX and PTX alone. In cells treated with drugs, intense green fluorescence of the Fluo-4 Direct™ probe and some changes in cell morphology, like cell shrinkage, were seen.

### 2.6. Mitochondrial Membrane Potential

**Figure 6:**
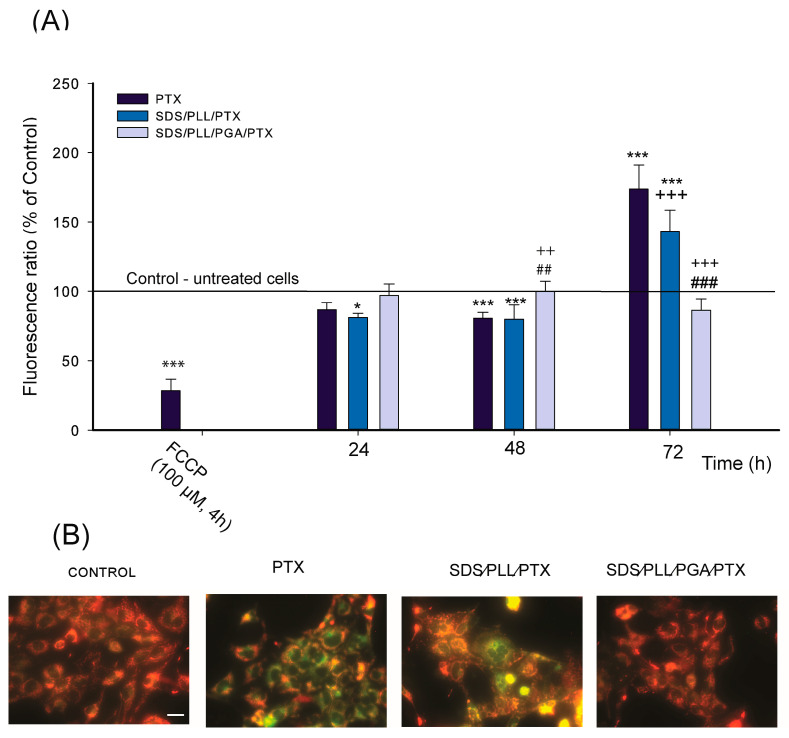
The impact of free PTX and PTX encapsulated in SDS-based nanocarriers on mitochondrial membrane potential in HL-1 Cells. (**A**) Mitochondrial membrane potential in HL-1 cells was assessed at 24, 48, and 72 h of treatment with the investigated anticancer compounds, using the JC-1 assay. The fluorescence ratio of JC-1 dimers to JC-1 monomers in control cells was set at 100%. Data are expressed as a mean ± SD from four independent experiments, normalized to control fluorescence. Statistical significance relative to control cells is indicated as * *p* < 0.05, *** *p* < 0.001, while ++ *p* < 0.01, +++ *p* < 0.001 denote significant differences between free PTX and encapsulated PTX formulations. Additionally, ## *p* < 0.01, ### *p* < 0.001 indicate significant differences between SDS/PLL/PTX and SDS/PLL/PGA/PTX-treated samples. (**B**) Representative fluorescence microscopy images of control cells treated with PBS and cells exposed to PTX-loaded nanocarriers at IC_50_ concentrations. Red fluorescence of JC-1 dimers indicates areas of high mitochondrial membrane potential, whereas green fluorescence of JC-1 monomers reflects areas of low mitochondrial membrane potential. Images were acquired using an Olympus IX70 inverted fluorescence microscope (Olympus, Tokyo, Japan) at 400× magnification.

The collapse of Δψm in HL-1 cardiomyocytes following treatment with the investigated PTX formulations for 24, 48, and 72 h was evaluated using the fluorescence probe JC-1. As a positive control (Figure 6A), an FCCP, a protonophoric uncoupler of oxidative phosphorylation, was used, resulting in a significant reduction in Δψm, evidenced by a decreased JC-1 dimer-to-monomer fluorescence ratio [29]. Free PTX and SDS/PLL/PTX induced a time-dependent reduction in Δψm, with the most pronounced decrease (~80%) observed after 48 h of treatment. After 72 h of incubation, a 1.7-fold and 1.4-fold reduction in JC-1 fluorescence intensity was recorded for free PTX and SDS/PLL/PTX, respectively. By contrast, SDS/PLL/PGA/PTX induced only a modest change, with a 1.2-fold reduction in the JC-1 dimer-to-monomer ratio after 72 h. The Δψm changes in cardiomyocytes were confirmed using fluorescence microscopy. As shown in Figure 6B, treatments with free PTX or SDS/PLL/PTX caused a marked increase in green JC-1 monomer fluorescence, indicative of reduced Δψm. Conversely, untreated cells and those exposed to SDS/PLL/PGA/PTX maintained high Δψm, evidenced by predominant red JC-1 dimer fluorescence.

### 2.7. Effects of PTX Encapsulated in SDS-Based NCs on the Expression of Genes Related to Cardiac Cell Homeostasis

**Figure 7:**
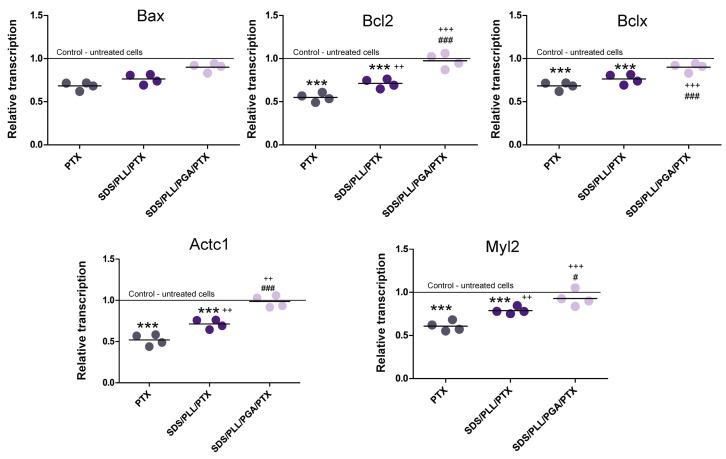
mRNA expression alterations of genes associated with apoptosis and cardiomyocytes function in HL-1 cells treated with SDS-based PTX nanocarriers and free PTX. The expression of Bax, Bcl-2, Bcl-xl, Myl2, and Actc1 was analyzed in HL-1 cardiomyocytes treated with SDS/PLL/PTX, SDS/PLL/PGA/PTX, or free PTX (at IC_50_ concentrations) for 48 h. Gene expression was quantified using average Ct values normalized to the housekeeping genes *β-actin* and *Gapdh*. Data were standardized to mRNA levels in untreated control cells, which were set to 1. Results are presented as a mean ± SD (*n* = 4). Statistically significant differences in gene expression between drug-treated and control cells are indicated as *** *p* < 0.001, while ++ *p* < 0.01, +++ *p* < 0.001 highlight significant differences between SDS/PLL/PTX, SDS/PLL/PGA/PTX, and free PTX-treated cells. Differences between SDS/PLL/PTX and SDS/PLL/PGA/PTX-treated samples are shown as # *p* < 0.01, ### *p* < 0.001.

Given the significant reduction in HL-1 cell metabolic activity induced by free PTX and SDS/PLL/PTX, we investigated their impact on the expression of genes involved in mitochondrial-dependent cell death pathways and cardiotoxicity markers. Expression of the anti-apoptotic genes *Bcl-2* and *Bcl-xl* was significantly downregulated in cells treated with SDS/PLL/PTX (1.4-fold) or free PTX (1.7-fold) (Figure 7). By contrast, the pro-apoptotic gene *Bax* was upregulated with expression increasing 2.4-fold and 1.7-fold for free PTX and SDS/PLL/PTX, respectively, compared to untreated controls. The SDS/PLL/PGA/PTX treatment did not alter the expression of these genes.

**Figure 8:**
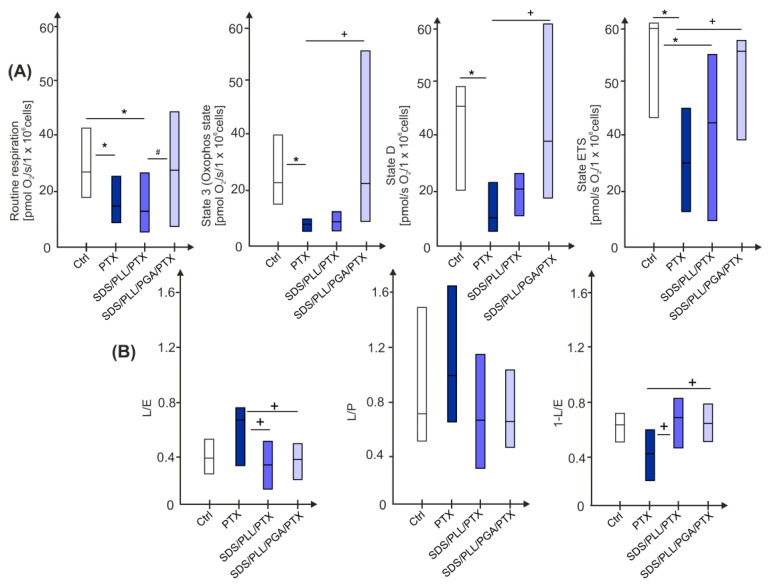
Differential effects of PTX and PTX encapsulated in SDS-based nanocarriers on mitochondrial function in HL-1 cells. (**A**) Mitochondrial respiration in HL-1 cardiomyocytes was evaluated following 72 h of treatment with IC_50_ concentrations of PTX, SDS/PLL/PTX, or SDS/PLL/PGA/PTX. Data are presented as the median oxygen consumption rate (pmol O_2_/s/1 × 10⁶ cells) measured in permeabilized cells. Parameters assessed include routine respiration, state 3 respiration, state D respiration, and electron transfer system (ETS) capacity. Significant differences relative to control of untreated cells are marked as * *p* < 0.05, while + *p* < 0.05 denotes differences between free PTX and SDS-based NCs. Comparisons between SDS/PLL/PTX and SDS/PLL/PGA/PTX-treated samples are marked as # *p* < 0.05. (**B**) Mitochondrial respiratory efficiency parameters, including L/E, L/P, and 1−L/E ratios, were determined in permeabilized HL-1 cells after 72 h of treatment with IC_50_ concentrations of free PTX or PTX encapsulated in SDS/PLL and SDS/PLL/PGA NCs. Statistical significance relative to untreated control cells is indicated as * *p* < 0.05. Significant differences between free PTX or encapsulated formulations of PTX are denoted as + *p* < 0.05, and differences between SDS/PLL/PTX and SDS/PLL/PGA/PTX-treated samples are marked as # *p* < 0.05.

To further assess cardiotoxicity, mRNA levels of *Myl2* and *Actc1*, genes associated with cardiac myosin cycling kinetics and actin polymerization, were analyzed. As shown in Figure 8, both genes were downregulated in cells treated with free PTX or SDS/PLL/PTX. The observed decrease in *Myl2* and *Actc1* transcript levels suggests that these treatments may contribute to cellular dysfunctions typically associated with idiopathic dilated cardiomyopathy (IDC). Notably, no significant changes in gene expression were observed in cells treated with SDS/PLL/PGA/PTX, underscoring its reduced impact on cardiac cell homeostasis.

### 2.8. Free PTX or Drug Encapsulated in SDS-Based Multicore Nanocarriers Alters Oxygen Consumption Rates of HL-1 Cardiomyocytes in a Different Way

**Figure 9:**
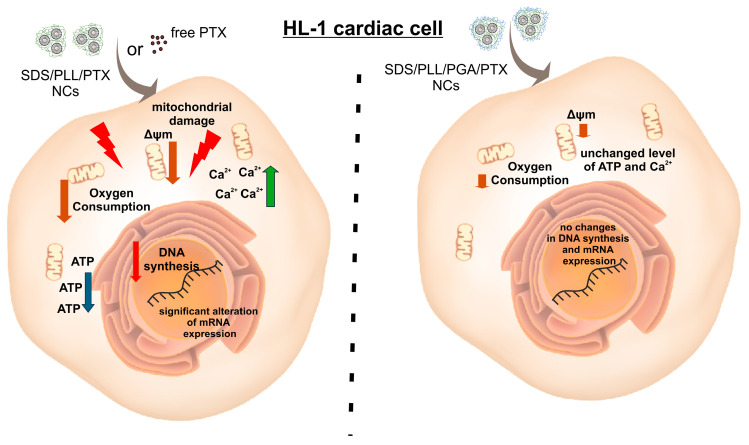
Simplified model illustrating the differential effects of free PTX or SDS/PLL/PTX versus SDS/PLL/PGA/PTX on HL-1 cardiomyocytes. Our findings show that free PTX or trapped in SDS/PLL NCs markedly depleted DNA synthesis (red arrow) and ATP levels (blue arrow), accompanied by a significant reduction in mitochondrial membrane potential and decrease in an oxygen consumption rate (brown arrows) and altered mRNA expression of genes associated with apoptosis and cardiomyocytes’ function. By contrast, the cytotoxic effects were less pronounced when PTX was encapsulated in SDS/PLL/PGA nanocarriers. In addition, this form of PTX demonstrated the lowest cytotoxicity towards cardiac cell cultures, with slim modification of cardiac cells’ homeostasis. Our finding indicates SDS/PLL/PGA/PTX potential as a safer formulation for reduction of the adverse effects of PTX on cardiomyocytes.

To investigate whether interactions of SDS/PLL/PTX, SDS/PLL/PGA/PTX, or free PTX with the HL-1 cardiomyocytes affect mitochondrial respiration, we monitored various mitochondrial parameters in digitonin permeabilized cells. Even though the experiment was performed after 24 and 48 h, the significant bioenergetic changes in HL-1 mitochondria were evident only after 72 h (Figure 8) of treatment with the tested forms of PTX. Firstly, we observed a significant difference in the mitochondrial routine respiration between cells treated with free PTX and those treated with SDS/PLL/PGA/PTX. As shown in Figure 9, a 2-fold reduction in initial respiration was noted for both free PTX (15.3 O_2_ pmol/s/1 × 10^6^ cells) and SDS/PLL/PTX (12.1 O_2_ pmol/s/1 × 10^6^ cells) in comparison to untreated, control cells.

Moreover, when the respiration of cardiomyocytes was restricted only to complexes 1 and 2 by the addition of sucrose, free PTX reduced mitochondrial respiration to half the value observed in untreated control cells (*p* < 0.05). To further assess mitochondrial function, we evaluated the cellular capacity to convert ADP to ATP (State D respiration). The slowest rate of ATP production was observed in cells treated with free PTX alone (Figure 9). Additionally, while evaluating the efficiency of the electron transport system (ETS) in the presence of free PTX or SDS/PLL/PTX, we found a 50% inhibition in the electron transport rate through the inner mitochondrial membrane. By contrast, SDS/PLL/PGA/PTX did not affect ETS efficiency. Next, we investigated the integrity of the mitochondrial membrane by calculating the respiratory control ratio (RCR). No significant differences in this parameter were observed among the tested groups. However, previous observations showed that free PTX and SDS-based PTX formulations altered Δψm. Thus, we calculated the leak control ratio (L/E), which describes the mitochondrial decoupling capacity. Interestingly, HL-1 cardiomyocytes exposed to SDS/PLL/PTX or SDS/PLL/PGA/PTX exhibited higher L/E values (0.626 and 0.606, respectively) compared to cells treated with free PTX alone (0.417), indicating an adaptive response to the nanocarrier formulations.

Finally, we assessed the ETS capacity efficiency, which reflects the overall performance of electron flow through the respiratory chain. The lowest values, with a 70% reduction, were observed in cells treated with free PTX alone (*p* = 0.026). By contrast, neither SDS/PLL/PTX nor SDS/PLL/PGA/PTX caused significant changes in the ETS efficiency. These results confirm that PTX encapsulated in SDS-based nanocarriers induces significantly less mitochondrial dysfunction compared to the free drug.

## 3. Discussion

Drug delivery systems (DDSs) are engineered to enhance drug efficacy while minimizing the off-target accumulation of therapeutic agents within the human body [16]. Nanotechnology offers a promising platform for advancing tumor diagnosis and treatment and providing sophisticated tools for anticancer therapy. The primary focus in nanotechnology is on nanoparticles (NPs), which exhibit immense potential in biomedical applications due to their high surface to volume ratio [30]. NPs can be specifically designed to improve drug distribution selectivity and overcome critical physiological barriers [31]. In particular, biodegradable polymeric NPs are emerging as highly promising carriers for active pharmaceutical compounds. Their utility in pharmacology lies in the ability to increase the therapeutic efficacy of drugs delivered in ‘nano’ form, while simultaneously reducing the risk of adverse side effects [32]. Encapsulation in NPs can improve drug solubility and stability, prolong circulation time, enhance bioavailability, and potentially lower the required therapeutic dose [33]. A versatile method for fabricating biodegradable polymeric is the sequential adsorption of oppositely charged polyelectrolytes, also known as the LbL technique [34]. Our previous research extensively investigated the cytotoxic properties of SDS-based multicore NCs loaded with PTX against breast cancer cells. We demonstrated that free PTX was less effective at inducing apoptosis compared to PTX encapsulated within SDS/PLL/PGA NCs [24].

Currently, in clinical settings, PTX is formulated as Taxol due to its low water solubility. Taxol is administered to patients as a mixture of Cremophor EL and dehydrated ethanol (50:50, *v*/*v*) [18]. However, Cremophor EL is not biologically inert and is associated with several adverse biological effects, including cardiovascular complications. Clinically, Taxol frequently induces asymptomatic reversible bradycardia, blood pressure fluctuations, arrhythmia, myocarditis, and pericarditis [35]. Moreover, the commonly used combination of PTX with anthracyclines has been shown to increase the risk of heart failure [36,37]. This effect is attributed to the prolonged presence of anthracyclines and their metabolites in vivo, which is exacerbated by PTX’s mechanism of action [38]. As a result, considerations of cardiac safety are critically important in the development of new cytostatic agents and their clinical application. To ensure the safety, health, and long-term efficacy of drugs, it is necessary to fully understand and evaluate the potential impact of these drugs on the heart. This requirement has led to the emergence of the field of cardio-oncology, which focuses on improving cardiovascular health in cancer patients [37]. Cardio-oncology aims to identify strategies that enable patients to maintain optimal cardiovascular health during and after treatment, thereby enhancing overall treatment outcomes. The scientific literature provides numerous examples of nanosystems developed to enhance PTX delivery while simultaneously reducing its cardiotoxicity. For instance, Abraxane, a nanoformulation of PTX approved by the FDA, has been successfully used for the treatment of breast cancer and advanced non-small cellular lung cancer. However, post-marketing surveillance has reported cases of congestive heart failure and left ventricular dysfunction in some individuals receiving Abraxane [39].

In this study, we focused on the evaluation of the cardiac safety of SDS/PLL and SDS/PLL/PGA NCs designed for PTX delivery directly to tumor tissue. Our primary aim was to assess the cardiovascular toxicity of newly synthesized SDS-based nanosystems loaded with PTX and to provide valuable references and guidelines for future research and clinical applications. We present evidence that exposition of HL-1 cardiac myocytes to PTX encapsulated in SDS-based NCs, particularly SDS/PLL/PGA/PTX, resulted in less pronounced disturbances to cellular homeostasis compared to treatment with free PTX (Figure 9). This conclusion is supported by comparisons of the cytotoxicity of the anticancer compounds, measurements of ATP levels, evaluations of Δψm, assessments of calcium ions leakage, and analyses of the expression of genes involved in the cardiac cell stress response. Our results clearly demonstrate that SDS/PLL/PGA/PTX exhibits lower toxicity to HL-1 cells than free PTX or SDS/PLL/PTX. Furthermore, the reduced toxicity of SDS/PLL/PGA/PTX correlated with observed changes in HL-1 cell morphology. When HL-1 cells were treated with IC_50_ concentrations of the tested drug formulations, cellular membrane disruption and fragmentation were primarily evident in cells treated with free PTX. The high cytotoxicity of PTX observed in vitro is consistent with previous findings. For instance, Balachandran et al. [18] reported a significant reduction (60.23%) in the viability of primary human cardiomyocytes treated with PTX. Additionally, our Hoechst staining analysis of DNA content revealed that neither SDS/PLL/PTX nor SDS/PLL/PGA/PTX exhibited as strong an antiproliferative effect as free PTX. The most significant reduction in DNA synthesis was observed when HL-1 cells were incubated with the tested PTX formulations for up to 72 h. These findings align with diminished ATP production measured using the CellTiter-Glo^®^ assay. Considering that ATP synthesis occurs in mitochondria, we investigated the impact of PTX encapsulated in SDS-based NCs on mitochondrial function in cardiac myocytes. Mitochondria, as bioenergetic and signaling organelles, play a crucial role in regulating apoptosis, metabolism, and cancer progression [40,41]. Additionally, mitochondria are indispensable in immune responses, rapid energy transduction, and other vital cellular processes [42]. Dysfunctional mitochondria can lead to muscle atrophy and diverse cardiovascular disease [13].

A common symptom of congestive heart failure is elevated cytosolic calcium levels, typically resulting from excessive calcium entry into or reduced calcium efflux from the cytosol [43]. It has been convincingly demonstrated that an increase in intracellular Ca^2+^ levels can act as a triggering event leading to cell damage [43]. For example, Kania et al. [44] showed that aclarubicin, an anthracycline anticancer agent, caused an elevation in the intracellular Ca^2+^ pool in both normal (S2) and trisomic (BB) diabetic fibroblasts. In our study, we observed that cellular Ca^2+^ levels increased following treatment with free PTX and SDS/PLL/PTX. This alteration was accompanied by a reduction in Δψm, which may suggest mitochondrial damage and the opening of the mitochondrial permeability transition pore (mPTP). The opening of mPTP facilitates the release of cytochrome c into the cytoplasm, ultimately leading to cell death. However, this process does not always correlate with a significant increase in reactive oxygen species (ROS) [45]. Our research (unpublished data) demonstrated that neither free PTX nor PTX encapsulated in SDS-based multicore NCs generated free radicals in noncancerous human endothelial cells. Thus, we hypothesize that the unaltered ROS levels observed following the PTX treatment do not inhibit the induction of mitochondrial damage. Hao et al. further described that free PTX alone increased mitochondrial mass in cells arrested in the M phase of the cell cycle [13]. In this context, we compared the mitochondrial dysfunction and energy stress triggered by PTX or drugs encapsulated in SDS-based nanocarriers. Following the Neuzil’s classification of anticancer agents that influence mitochondrial homeostasis [46], we investigated whether there is a direct relationship between mitochondrial functional disturbances and the form of tested drugs. Our findings confirmed that the first detectable markers of disorders in mitochondrial respiration in HL-1 cardiomyocytes appeared only after 72 h of incubation. No significant changes were observed after 24 or 48 h of treatment. Interestingly, after 72 h, PTX-treated cells exhibited a ~50% increase in MMP, suggesting hyperpolarization of the inner mitochondrial membrane. Additionally, data from the monitoring of ADP conversion into ATP revealed that the slowest rate of ATP production occurred in cells exposed to free PTX. Furthermore, the efficiency of the electron transfer system was significantly reduced in HL-1 cells treated with PTX or SDS/PLL/PTX.

Alterations in mitochondrial bioenergetics were closely linked to changes in the expression of genes involved in maintaining the homeostasis of cardiac cells. For instance, a significant decrease in the mRNA levels of anti-apoptotic genes *Bcl2* and *Bcl-xl* was predominantly observed in PTX-treated samples, alongside an increase in the expression of the pro-apoptotic gene *Bax*. Additionally, incubation with free PTX corresponded with the downregulation of genes involved in actin and myosin formation, such as *Myl2* and *Actc*. Myosin light chain (MLC) plays a crucial role in myocardial contraction, diastole, cardiac electrophysiological homeostasis, vascular nerve vasoconstriction, and blood pressure regulation. Similarly, actin forms the thin filaments of myofibrils and contributes to maintaining cell shape and movement as part of the cytoskeleton [47]. The interaction between myosin and actin promotes muscle contraction via the phosphorylation of myosin regulatory light chain 9 (MYL9), which enhances MLC ATPase activity and facilitates cell movement. In this study, neither SDS/PLL/PTX nor SDS/PLL/PGA/PTX significantly disrupted cardiac cell homeostasis compared to free PTX. These observations aligned with an increased number of shrunken cells and reduced cell proliferation in PTX-treated samples.

Why did SDS-based NCs loaded with PTX exhibit diverse cytotoxicity towards cardiac myocytes? It has been proposed that polyelectrolyte drug nanocarriers can preferentially accumulate in tumors due to enhanced vascular permeability and impaired lymphatic drainage in cancer cells [48,49]. However, interactions between newly developed nanosystems and non-cancerous cells, as well as the sensitivity of healthy tissues to their antiproliferative effects, remain poorly understood. Numerous studies have demonstrated that suspensions of nanoparticles in biological fluids undergo rapid aggregation. This phenomenon occurs due to the disparity in ionic strength between biological fluids and nanostructures or the adsorption of serum proteins onto the administered nanomaterials. Therefore, to reduce the risk of adverse interactions between nanocarriers and biological fluids, the surfaces of nanomaterials are coated with hydrophilic, flexible, and non-ionic polyelectrolytic polymers. Polyelectrolytes employed in the LbL technique, such as those described here, poly-L-lysine (PLL) and poly-L-glutamic acid (PGA) exhibit properties similar to those of biomacromolecules that readily dissociate in aqueous solutions [50,51]. In addition, due to their relatively simple synthesis and ease of purification, these polyelectrolytes, when used to form nanocapsule coatings, are characterized by high biodegradability, low toxicity, and minimal potential to alter the cytotoxicity of the transported drugs.

During the biodegradation process, the by-products of NCs are biocompatible or harmless [52]. However, the polyester bonds within the nanocarrier’s structure remain stable in the blood circulation and extracellular fluid [53,54]. Once internalized by cells, these bonds are rapidly cleaved under intracellular conditions, allowing for the release of PTX from the NCs. The oppositely charged polyelectrolyte layers covering the micellar core provide long-term colloidal stability and prevent aggregation. Moreover, the use of SDS as a water-soluble surfactant is vital for achieving high loading capacity and physical stability of the multifunctional cargo. These factors support the preclinical evaluation of SDS-based nanocarriers designed for medical imaging and personalized cancer therapy. The novel multicore polyelectrolyte NCs developed here can carry both hydrophobic and hydrophilic drugs, thus facilitating the delivery of unique drug combinations crucial for personalized medicine.

The response of murine HL-1 cardiomyocytes to different forms of PTX appears to be influenced by their unique modes of cellular transport. One notable distinction among the tested NCs is their charge. Numerous studies have shown that positively charged nanoparticles are internalized more efficiently by cells [55,56,57]. This phenomenon can be attributed to the negatively charged nature of cellular membranes, which facilitates the uptake of positively charged SDS/PLL NCs via electrostatic attraction. Understanding these mechanisms could provide valuable insights for optimizing the design of nanocarriers for cancer therapy in the near future.

In conclusion, we demonstrated for the first time that SDS/PLL/PGA NCs reduced the cytotoxicity of PTX released from the carrier towards cardiac myocytes. Compared to the free form of PTX or SDS/PLL/PTX, there was no remarkable decrease in the oxygen consumption rate in samples treated with SDS/PLL/PGA/PTX. The expression of genes involved in actin and myosin formation was altered when HL-1 cardiomyocyte cells were exposed to PTX alone or encapsulated in SDS/PLL NCs. As a promising drug delivery system, SDS/PLL/PGA/PTX caused no significant changes in ATP level, calcium concentration, and mitochondrial membrane potential. These preclinical data suggest that SDS/PLL/PGA NCs could be beneficial in multimodal therapy settings based on PTX application.

## 4. Materials and Methods

### 4.1. Reagents

PTX was supplied by Selleckchem (Houston, TX, USA), Claycomb medium, fetal bovine serum (FBS), gelatin, fibronectin, glutamine, norepinephrine, penicillin, streptomycin, dimethyl sulfoxide (DMSO), malate, glutamate, succinate, rotenone, oligomycin, antimycin A, digitonin, ADP, carbonyl cyanide-p-trifluoromethoxyphenylhydrazone, 3-(4,5-dimethyl-2-thiazolyl)-2,5-diphenyl-2H-tetrazolium bromide (MTT), 5,50,6,60-tetrachloro-1,10,3,30-tetraethyl-benzimidazolcarbocyanineiodide (JC-1), Hoechst 33258, chlorophenylhydrazone (FCCP), sodium chloride (NaCl), phosphate buffered saline (PBS) and all reagents for carrying out the nanocapsule synthesis (sodium dodecyl sulphate (SDS), poly-L-lysine hydrobromide (PLL, MW 15,000–30,000), poly-L-glutamic acid sodium salt (PGA, MW 15,000–50,000)) were obtained from Sigma-Aldrich (Darmstadt, Germany). Fluo-4 Direct™, resazurin sodium salt (7-Hydroxy-3H-phenoxazin-3-one-10-oxide sodium salt), Hank’s balanced salt solution (HBSS), Maxima First Strand cDNA Synthesis Kit for RT-qPCR were supplied by Thermo Fisher Scientific (Waltham, MA, USA), while CellTiter-Glo^®^ assay was supplied by Promega (Walldorf, Germany). The SYBR-green PCR master mix was provided by Roche (Basel, Switzerland). Tissue culture flasks and plates were supplied by TPP (Trasadingen, Switzerland). Other chemicals and solvents of high analytical grade were purchased from POCH S.A. (Gliwice, Poland). PTX was encapsulated into SDS-based nanocapsules using the modified LbL procedure. The obtained NCs (Figure 1A) were characterized by the determination of particle size, shape, and zeta potential [24]. All details related to the synthesis and physico-chemical properties of SDS/PLL or SDS/PLL/PGA nanosystems (empty or loaded with drug) were described in patent claim no WIPO ST 10/C PL443843.

### 4.2. Preparation and Characterization of SDS-Based NCs

Four variants of SDS-based multicore nanocarriers (NCs) were prepared using the layer-by-layer (LbL) assembly method as previously described [24]. The prepared NCs were characterized using dynamic light scattering (DLS) and electrophoretic light scattering (ELS) techniques with a Zetasizer Nano ZS instrument (Malvern Instruments, Malvern, UK).

### 4.3. Cell Culture

HL-1 cardiomyocytes, generously provided by Professor W.C. Claycomb (Louisiana State University, New Orleans, LA, USA), were cultured on a matrix composed of 0.02% (*w*/*v*) gelatin and 10 μg/mL fibronectin. The cells were maintained in a Claycomb medium supplemented with 10% (*v*/*v*) fetal bovine serum (FBS), 2 mM L-glutamine, 0.1 mM norepinephrine, 100 U/mL penicillin, and 100 U/mL streptomycin. Cells were incubated under standard culture conditions at 37 °C with 100% humidity in an atmosphere of 5% CO2 and 95% air. For all experiments, cells in the logarithmic growth phase were utilized to ensure consistency and reliability.

### 4.4. DNA Content Estimation

HL-1 cells were seeded into 96-well black plates (TPP, Trasadingen, Switzerland) at densities of 4000, 7000, or 10,000 cells per well, depending on the experimental time point. The following day, cells were treated with PTX, SDS/PLL/PTX, or SDS/PLL/PGA/PTX at IC_50_ concentrations in fresh growth medium. Incubation with the compounds continued for 24, 48, or 72 h at 37 °C. After the treatment period, the medium was aspirated, and the cell monolayers were frozen at −70 °C. The cultures were then thawed at room temperature and deionized water (100 µL) was added to each well. The plates were frozen a second time at −70 °C and subsequently thawed. Following thawing, 100 µL of Hoechst 33258 solution (1 µmol/dm³ in a TE buffer; 10 mM Tris-HCl, 1 mM EDTA) was added to each well [58]. Plates were immediately shaken and incubated for 15 min in the dark at room temperature. Fluorescence measurements were performed at an excitation/emission wavelength of 355/460 nm using a Fluoroskan Ascent FL microplate reader (Labsystems, Farsta, Sweden). Fluorescence intensity values for control plates were normalized to 100%.

### 4.5. Cell Viability Assays

The viability of HL-1 cardiomyocytes exposed to SDS-based nanocarriers (NCs), both drug-loaded and empty, was assessed using the MTT assay and the resazurin reduction assay. Morphological changes in cells were also analyzed via microscopy (Olympus IX70, Tokyo, Japan). The percentage of viable cells was calculated by comparing the fluorescence (Alamar Blue assay) or absorbance (MTT assay) of treated cells with untreated control cells [59]. The cytotoxicity of paclitaxel (PTX) and its encapsulated forms (SDS/PLL/PTX or SDS/PLL/PGA/PTX) was expressed as the IC_50_ value, defined as the concentration of the agent required to reduce cell viability by 50% compared to untreated controls. IC_50_ values were calculated using GraphPad Prism 4.03 (GraphPad Software, Boston, MA, USA).

#### 4.5.1. MTT Assay

HL-1 cells (4000 cells/well) were seeded in 96-well flat-bottomed microtiter plates (TPP, Trasadingen, Switzerland) in 100 μL of culture medium. After 24 h, the cells were exposed in triplicate to varying concentrations of SDS/PLL/PTX, SDS/PLL/PGA/PTX, or free PTX for 72 h under standard culture conditions (37 °C, 5% CO_2_, 100% humidity). Parallel experiments were performed with empty NCs (SDS/PLL or SDS/PLL/PGA) as controls. Following incubation, the medium was aspirated, and 50 μL of an MTT solution (50 mg/100 mL, Sigma-Aldrich, Darmstadt, Germany) was added to each well. After 3 h, the resulting formazan crystals were dissolved in DMSO, and the plate was gently shaken for 60 s [60]. Absorbance was measured at 580 nm and 720 nm using a spectrophotometric plate reader (Awareness Technology Inc., Palm City, FL, USA).

#### 4.5.2. Resazurin Reduction Assay

HL-1 cells were seeded into 96-well black plates (TPP, Trasadingen, Switzerland) at a density of 4000 cells per well one day prior to treatment. The cells were then incubated with various concentrations of test compounds for 72 h at 37 °C. Following incubation, 100 μL of a resazurin solution (0.0125 mg/mL in a medium without phenol red and FBS) was added to each well [59]. The plate was then returned to standard culture conditions for 3 h. Fluorescence was measured using a spectrofluorometric plate reader (Fluoroskan Ascent FL, Labsystems, Farsta, Sweden) with an excitation wavelength of 530 nm and an emission wavelength of 590 nm.

#### 4.5.3. Mitochondrial Membrane Potential Analysis

The mitochondrial membrane potential (ΔΨm) was analyzed using the cationic dye JC-1 (Sigma-Aldrich, Darmstadt, Germany), which selectively labels mitochondria in living cells [61]. HL-1 cells in the logarithmic growth phase were treated with IC_50_ concentrations of PTX, SDS/PLL/PTX, or SDS/PLL/PGA/PTX for 24, 48, and 72 h. FCCP (final concentration: 100 μM), a known uncoupler of oxidative phosphorylation, was used as a positive control for mitochondrial depolarization. At the end of the treatment period, the culture medium was removed, and JC-1 probe solution (5 μM) was added to the cells, which were incubated for 30 min. Following incubation, the probe was removed, and cells were rinsed with DMEM without phenol red and FBS. DMEM (50 μL) was then added to each well. Fluorescence was measured using a Fluoroskan Ascent plate reader (Labsystems, Farsta, Sweden) with filter pairs of 530 nm/590 nm and 485 nm/538 nm [62]. The ΔΨm results are presented as the ratio of fluorescence intensity at 530 nm/590 nm (JC-1 dimers) to fluorescence at 485 nm/538 nm (JC-1 monomers). This ratio was normalized to the control of untreated cells, whose ratio was set at 100%. In parallel the photos were acquired using fluorescence microscope equipped with digital camera (Olympus IX70, Tokyo, Japan).

### 4.6. ATP Measurements

The ATP levels in cardiac cells treated with investigated compounds were quantified luminometrically using the CellTiter-Glo^®^ assay (Promega, Walldorf, Germany). Cells were seeded into white 96-well plates (TPP, Trasadingen, Switzerland) and incubated with the tested PTX formulations for 24, 48, and 72 h at 37 °C. After the treatment period, half of the medium volume was removed and replaced with an equal volume of the ATP detection reagent. The plate was gently mixed on an orbital shaker for 10 min to ensure complete cell lysis [63]. Subsequently, 100 μL from each well was transferred to a separate white plate, and luminescence was measured using a Fluoroskan Ascent plate reader (Labsystems, Farsta, Sweden) without an emission filter.

### 4.7. Intracellular Calcium Measurement

Intracellular calcium levels were assessed in HL-1 cells cultured in black fluorometric 96-well plates and treated with PTX, SDS/PLL/PTX, or SDS/PLL/PGA/PTX. Thapsigargin (5 μM for 30 min; Sigma-Aldrich, Darmstadt, Germany) was used as a positive control. After the treatment, the growth medium was aspirated, and cells were washed with PBS. A 100 μL volume of Fluo-4 Direct™ dye working solution, prepared following the manufacturer’s instructions (Thermo Fisher Scientific, Waltham, MA, USA), was added to each well. The plate was incubated in the dark at 37 °C for 30 min and subsequently for an additional 30 min at room temperature [44]. Fluorescence was measured on a Fluoroskan Ascent FL microplate reader (Labsystems, Farsta, Sweden) using 494 nm excitation and 516 nm emission wavelengths. In parallel the photos were acquired using fluorescence microscope equipped with digital camera (Olympus IX70, Tokyo, Japan).

### 4.8. High-Resolution Respirometry

**Figure 10:**
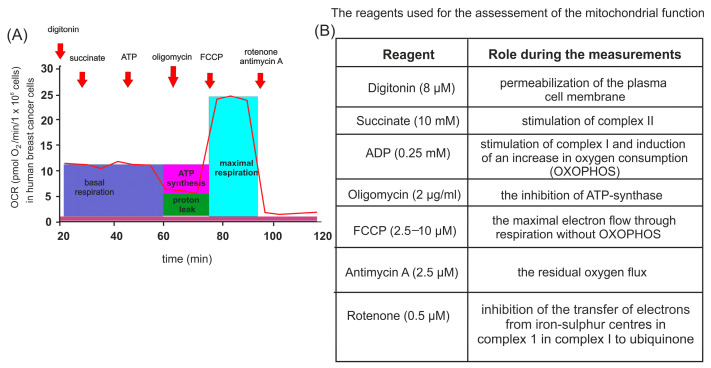
Analytical setup for high-resolution spirometry of HL-1 cells and assessment of cellular oxygen consumption. (**A**) Schematic representation of the sequential addition of substrates during oxygen consumption rate (OCR) measurements. (**B**) Overview of the rationale for using specific chemical compounds in mitochondrial bioenergetics analysis.

Mitochondrial bioenergetics in HL-1 cardiomyocytes treated with PTX, SDS/PLL/PTX, or SDS/PLL/PGA/PTX was analyzed using an Oroboros-2k oxygraph (Oroboros Instruments, Innsbruck, Austria). The experiments were conducted at 37 °C in a MIRO5 medium (110 mM sucrose, 60 mM K-lactobionate, 0.5 mM EGTA, 1 g/L BSA, 3 mM MgCl_2_, 20 mM taurine, 10 mM KH_2_PO_4_, and 20 mM HEPES, pH 7.0) under continuous stirring at 750 rpm. Substrates and inhibitors required for mitochondrial oxidation (Figure 10A,B) were used [64]. Respiration rates were expressed as picomoles O_2_ per second per milligram of wet weight. Briefly, cells (0.05 mL) were resuspended in a MIRO5 buffer, added to each chamber, and stabilized under routine respiration conditions. Permeabilization of the plasma membrane was achieved by adding digitonin (10 μg/mL), and succinate (10 mM) was then introduced to stimulate respiration via complex II. To determine the involvement of State 4 respiration, maximal oxidative phosphorylation (OXPHOS) was induced with ADP (0.25 mM), and ATP synthase was subsequently inhibited with oligomycin (2 μg/mL). The ETS state, indicating maximal electron flow through the respiratory chain in the absence of OXPHOS, was evaluated by FCCP titration (2.5–10 μM). Uncoupled complex I-linked respiration was assessed using rotenone (0.5 μM), and the ETS was finally inhibited with antimycin A (2.5 μM) to measure residual oxygen flux (ROX). The coupling efficiency of phosphorylation to oxidation was expressed as the respiratory control ratio (RCR), calculated as the ratio of ADP-stimulated respiration to basal respiration (without ADP), using glutamate and malate as substrates [65]. Data acquisition and analysis were performed with Datlab4 software (Oroboros, Innsbruck, Austria).

### 4.9. Quantitative Real-Time RT-PCR

Total RNA was extracted from cells treated with PTX, SDS/PLL/PTX, or SDS/PLL/PGA/PTX (at IC_50_ concentrations) for up to 48 h using TRI Reagent (Sigma, St. Louis, MO, USA) in accordance with the manufacturer’s protocol. RNA concentration and purity were assessed using a NanoDrop™ 2000 UV–Vis spectrophotometer (Thermo Fisher Scientific, Waltham, MA, USA). cDNA was synthesized using the Maxima First Strand cDNA Synthesis Kit for RT-qPCR (Thermo Fisher Scientific). Quantitative real-time PCR was carried out using SYBR Green PCR Master Mix (Roche, Basel, Switzerland) on a LightCycler^®^ 480 (Roche) [24]. PCR parameters included an initial denaturation at 94 °C for 4 min, followed by 40 cycles of denaturation at 94 °C for 15 s, annealing at 60 °C for 25 s, and extension at 72 °C for 25 s. Gene expression levels were normalized to *β-actin* and Glyceraldehyde-3-Phosphate Dehydrogenase (*Gapdh*). Primer sequences used were as follows: B-cell lymphoma 2 (*Bcl-2*) forward, 5′-GTGGATGACTGAGTACCT-3′ and reverse, 5′-CCAGGAGAAATCAAACAGAG-3′; Bcl-2-like protein 4 (*Bax*) forward, 5′-CTACAGGGTTTCATCCAG -3, and reverse, 5′-CCAGTTCATCTCCAATTCG-3; B-cell lymphoma-extra-large (*Bcl-xl*) forward, 5′-TGGAGTAAACTGGGGGTCGCATCG-3′, and reverse, 5′-AGCCACCGTCATGCCCGTCAGG-3′; myosin regulatory light chain 2 (*Myl2*) forward, 5′-AAGGACTGAGCCCTGAACCA-3′, and reverse, 5′-ACAGCCCTGGGATGGAGAGT-3′; actin alpha cardiac muscle 1 (*Actc1*) forward, 5′-CCAAAGCTGTGCCAGGATGT-3′, and reverse, 5′-GCCATTGTCACACACCAAAGC-3′; *β-actin* forward, 5′-GTGACGTTGACATCCGTAAAGA-3′, and reverse, 5′-GTAACAGTCCGCCTAGAAGCAC-3′; *Gapdh* 5′-CATCACTGCCACCCAGAAGA-3′ and reverse 5′-GCTGTAGCCAAATTCGTTGT-3′. Relative gene expression was calculated as the mean ΔCt normalized to housekeeping genes *Gapdh* and *β-actin*.

### 4.10. Statistical Analysis

Data are presented as a mean ± SD (*n* ≥3). The normality of data distribution was assessed using the Shapiro–Wilk test, and homogeneity of variance was evaluated with the Brown–Forsythe test. Differences between samples were determined using multivariate analysis of variance (ANOVA), followed by Tukey’s post hoc test for pairwise comparisons. Statistical analyses were performed using STATISTICA 11 (StatSoft, Tulsa, OK, USA). IC_50_ calculations were conducted with GraphPad Prism 5.0 (GraphPad Software, Boston, USA), and graphs were prepared using SIGMA-PLOT 12.0 (Systat Software Inc., San Jose, USA). A *p*-value of <0.05 was considered statistically significant.

## 5. Patents

Patent claim no. WIPO ST 10/C PL443843.

## Data Availability

The datasets presented during the current study are available from the corresponding author on reasonable request.

## References

[1] Weaver K.E., Foraker R.E., Alfano C.M., Rowland J.H., Arora N.K., Bellizzi K.M., Hamilton A.S., Oakley-Girvan I., Keel G., Aziz N.M. (2013). Cardiovascular risk factors among long-term survivors of breast, prostate, colorectal, and gynecologic cancers: A gap in survivorship care?. J. Cancer Surviv..

[2] Chen D., Kelly C., Haw T.J., Lombard J.M., Nordman I.I.C., Croft A.J., Ngo D.T.M., Sverdlov A.L. (2021). Heart Failure in Breast Cancer Survivors: Focus on Early Detection and Novel Biomarkers. Curr. Heart Fail. Rep..

[3] Du X., Khan A.R., Fu M., Ji J., Yu A., Zhai G. (2018). Current development in the formulations of non-injection administration of paclitaxel. Int. J. Pharm..

[4] Balachandran L., Haw T.J., Leong A.J.W., Croft A.J., Chen D., Kelly C., Sverdlov A.L., Ngo D.T. (2024). Cancer Therapies and Cardiomyocyte Viability: Which Drugs are Directly Cardiotoxic?. Heart Lung Circ..

[5] Titlyanov E., Titlyanova T., Yamazato K., van Woesik R. (2001). Photo-acclimation of the hermatypic coral *Stylophora pistillata* while subjected to either starvation or food provisioning. J. Exp. Mar. Biol. Ecol..

[6] Zhang K., Heidrich F.M., DeGray B., Boehmerle W., Ehrlich B.E. (2010). Paclitaxel accelerates spontaneous calcium oscillations in cardiomyocytes by interacting with NCS-1 and the InsP3R. J. Mol. Cell. Cardiol..

[7] Zhou M., Han S., Aras O., An F. (2021). Recent Advances in Paclitaxel-based Self-Delivery Nanomedicine for Cancer Therapy. Curr. Med. Chem..

[8] Abu Samaan T.M., Samec M., Liskova A., Kubatka P., Büsselberg D. (2019). Paclitaxel’s Mechanistic and Clinical Effects on Breast Cancer. Biomolecules.

[9] Alves R.C., Fernandes R.P., Eloy J.O., Salgado H.R.N., Chorilli M. (2018). Characteristics, Properties and Analytical Methods of Paclitaxel: A Review. Crit. Rev. Anal. Chem..

[10] Tung N.-T., Tran C.-S., Pham T.-M., Nguyen H.-A., Nguyen T.-L., Chi S.-C., Nguyen D.-D., Bui T.-B. (2018). Development of solidified self-microemulsifying drug delivery systems containing L-tetrahydropalmatine: Design of experiment approach and bioavailability comparison. Int. J. Pharm..

[11] Morelli M.B., Bongiovanni C., Da Pra S., Miano C., Sacchi F., Lauriola M., D’uva G. (2022). Cardiotoxicity of Anticancer Drugs: Molecular Mechanisms and Strategies for Cardioprotection. Front. Cardiovasc. Med..

[12] Volkova M., Russell R. (2011). Anthracycline cardiotoxicity: Prevalence, pathogenesis and treatment. Curr. Cardiol. Rev..

[13] Hao X., Bu W., Lv G., Xu L., Hou D., Wang J., Liu X., Yang T., Zhang X., Liu Q. (2022). Disrupted mitochondrial homeostasis coupled with mitotic arrest generates antineoplastic oxidative stress. Oncogene.

[14] Fukuta T., Ikeda-Imafuku M., Iwao Y. (2023). Development of Edaravone Ionic Liquids and Their Application for the Treatment of Cerebral Ischemia/Reperfusion Injury. Mol. Pharm..

[15] Choi B., Park W., Park S.-B., Rhim W.-K., Han D.K. (2020). Recent trends in cell membrane-cloaked nanoparticles for therapeutic applications. Methods.

[16] Huang L., Zhao S., Fang F., Xu T., Lan M., Zhang J. (2021). Advances and perspectives in carrier-free nanodrugs for cancer chemo-monotherapy and combination therapy. Biomaterials.

[17] Chehelgerdi M., Chehelgerdi M., Allela O.Q.B., Pecho RD C., Jayasankar N., Rao D.P., Thamaraikani T., Vasanthan M., Viktor P., Lakshmaiya N. (2023). Progressing nanotechnology to improve targeted cancer treatment: Overcoming hurdles in its clinical implementation. Mol. Cancer.

[18] Jiang Y., Jiang Y., Li M., Yu Q. (2023). Will nanomedicine become a good solution for the cardiotoxicity of chemotherapy drugs?. Front. Pharmacol..

[19] Yang J.-X., Yang Y.-Q., Hu W.-Y., Yang L., Wu J., Wen X.-X., Yu J., Huang M.-L., Xu D.-D., Tie D.-C. (2024). A Phase II Study of Neoadjuvant PLD/Cyclophosphamide and Sequential nab-Paclitaxel Plus Dual HER2 Blockade in HER2-Positive Breast Cancer. Oncologist.

[20] Nam S.H., Lee S.-W., Lee Y.-J., Kim Y.M. (2023). Safety and Tolerability of Weekly Genexol-PM, a Cremophor-Free Polymeric Micelle Formulation of Paclitaxel, with Carboplatin in Gynecologic Cancer: A Phase I Study. Cancer Res. Treat..

[21] Liu T., Wang Y., Zhong W., Li B., Mequanint K., Luo G., Xing M. (2019). Biomedical Applications of Layer-by-Layer Self-Assembly for Cell Encapsulation: Current Status and Future Perspectives. Adv. Healthc. Mater..

[22] Hashemi M., Omidi M., Muralidharan B., Tayebi L., Herpin M.J., Mohagheghi M.A., Mohammadi J., Smyth H.D., Milner T.E. (2018). Layer-by-layer assembly of graphene oxide on thermosensitive liposomes for photo-chemotherapy. Acta Biomater..

[23] Szczepanowicz K., Hoel H.J., Szyk-Warszynska L., Bielańska E., Bouzga A.M., Gaudernack G., Simon C., Warszynski P. (2010). Formation of biocompatible nanocapsules with emulsion core and pegylated shell by polyelec-trolyte multilayer adsorption. Langmuir.

[24] Szwed M., Michlewska S., Kania K., Szczęch M., Marczak A., Szczepanowicz K. (2023). New SDS-Based Polyelectrolyte Multicore Nanocarriers for Paclitaxel Delivery-Synthesis, Characterization, and Activity against Breast Cancer Cells. Cells.

[25] Zhu S., Wang X., Jing C., Yin Y., Zhou N. (2019). A colorimetric ATP assay based on the use of a magnesium(II)-dependent DNAzyme. Microchim. Acta.

[26] Ligasová A., Koberna K. (2019). Quantification of fixed adherent cells using a strong enhancer of the fluorescence of DNA dyes. Sci. Rep..

[27] Viskupicova J., Rezbarikova P. (2022). Natural Polyphenols as SERCA Activators: Role in the Endoplasmic Reticulum Stress-Related Diseases. Molecules.

[28] Makio T., Chen J., Simmen T. (2024). ER stress as a sentinel mechanism for ER Ca^2+^ homeostasis. Cell Calcium.

[29] Samartsev V.N., Belosludtsev K.N., Pavlova E.K., Pavlova S.I., Semenova A.A., Dubinin M.V. (2024). Theoretical and Experimental Study of the Interaction of Protonophore Uncouplers and Decoupling Agents with Functionally Active Mitochondria. Cell Biochem. Biophys..

[30] van der Meel R., Sulheim E., Shi Y., Kiessling F., Mulder W.J., Lammers T. (2019). Smart cancer nanomedicine. Nat. Nanotechnol..

[31] Mitchell M.J., Billingsley M.M., Haley R.M., Wechsler M.E., Peppas N.A., Langer R. (2021). Engineering precision nanoparticles for drug delivery. Nat. Rev. Drug Discov..

[32] Wu X., Wang X., Zhang H., Chen H., He H., Lu Y., Tai Z., Chen J., Wu W. (2024). Enhanced in vivo Stability and Antitumor Efficacy of PEGylated Liposomes of Paclitaxel Palmitate Prodrug. Int. J. Nanomed..

[33] Blanco E., Shen H., Ferrari M. (2015). Principles of nanoparticle design for overcoming biological barriers to drug delivery. Nat. Biotechnol..

[34] Elizarova I.S., Luckham P.F. (2018). Layer-by-layer adsorption: Factors affecting the choice of substrates and polymers. Adv. Colloid Interface Sci..

[35] Gelderblom H., Verweij J., Nooter K., Sparreboom A. (2001). Cremophor EL: The drawbacks and advantages of vehicle selection for drug formulation. Eur. J. Cancer.

[36] Agunbiade T.A., Zaghlol R.Y., Barac A. (2019). Heart Failure in Relation to Anthracyclines and Other Chemotherapies. Methodist DeBakey Cardiovasc. J..

[37] Bhagat A., Kleinerman E.S. (2020). Anthracycline-Induced Cardiotoxicity: Causes, Mechanisms, and Prevention. Adv. Exp. Med. Biol..

[38] Mondal P., Jain D., Aronow W.S., Frishman W.H. (2019). Cardiotoxicity of Cancer Therapies. Cardiol. Rev..

[39] Hirsh V. (2014). nab-paclitaxel for the management of patients with advanced non-small-cell lung cancer. Expert Rev. Anticancer. Ther..

[40] Wang D., Yi H., Geng S., Jiang C., Liu J., Duan J., Zhang Z., Shi J., Song H., Guo Z. (2023). Photoactivated DNA Nanodrugs Damage Mitochondria to Improve Gene Therapy for Reversing Chemoresistance. ACS Nano.

[41] Liu Y., Sun Y., Guo Y., Shi X., Chen X., Feng W., Wu L.-L., Zhang J., Yu S., Wang Y. (2023). An Overview: The Diversified Role of Mitochondria in Cancer Metabolism. Int. J. Biol. Sci..

[42] Yaqoob M.D., Xu L., Li C., Leong M.M.L., Xu D.D. (2022). Targeting mitochondria for cancer photodynamic therapy. Photodiagnosis Photodyn. Ther..

[43] Gorski P.A., Ceholski D.K., Hajjar R.J. (2015). Altered myocardial calcium cycling and energetics in heart failure—A rational approach for disease treatment. Cell Metab..

[44] Kania K., Zych A., Jóźwiak Z. (2007). Involvement of reactive oxygen species in aclarubicin-induced death of human trisomic and diabetic fibroblasts. Toxicol. In Vitro.

[45] Bernardi P., Gerle C., Halestrap A.P., Jonas E.A., Karch J., Mnatsakanyan N., Pavlov E., Sheu S.-S., Soukas A.A. (2023). Identity, structure, and function of the mitochondrial permeability transition pore: Controversies, consensus, recent advances, and future directions. Cell Death Differ..

[46] Neuzil J., Dong L.-F., Rohlena J., Truksa J., Ralph S.J. (2013). Classification of mitocans, anti-cancer drugs acting on mitochondria. Mitochondrion.

[47] Ran Q., Li A., Tan Y., Zhang Y., Zhang Y., Chen H. (2024). Action and therapeutic targets of myosin light chain kinase, an important cardiovascular signaling mechanism. Pharmacol. Res..

[48] Hinz A., Szczęch M., Szczepanowicz K., Bzowska M. (2022). Fluorophore Localization Determines the Results of Biodistribution of Core-Shell Nanocarriers. Int. J. Nanomed..

[49] Szczęch M., Hinz A., Łopuszyńska N., Bzowska M., Węglarz W.P., Szczepanowicz K. (2021). Polyaminoacid Based Core@shell Nanocarriers of 5-Fluorouracil: Synthesis, Properties and Theranostics Application. Int. J. Mol. Sci..

[50] Szczęch M., Szczepanowicz K. (2020). Polymeric Core-Shell Nanoparticles Prepared by Spontaneous Emulsification Solvent Evaporation and Functionalized by the Layer-by-Layer Method. Nanomaterials.

[51] Karabasz A., Bzowska M., Szczepanowicz K. (2020). Biomedical Applications of Multifunctional Polymeric Nanocarriers: A Review of Current Literature. Int. J. Nanomed..

[52] Ziąbka M., Dziadek M. (2019). Long-Term Stability of Two Thermoplastic Polymers Modified with Silver Nanoparticles. Nanomaterials.

[53] Drozdek S., Bazylińska U. (2016). Biocompatible oil core nanocapsules as potential co-carriers of paclitaxel and fluorescent markers: Preparation, characterization, and bioimaging. Colloid Polym. Sci..

[54] Geszke-Moritz M., Moritz M. (2024). Biodegradable Polymeric Nanoparticle-Based Drug Delivery Systems: Comprehensive Overview, Perspectives and Challenges. Polymers.

[55] Nam J., Won N., Bang J., Jin H., Park J., Jung S., Jung S., Park Y., Kim S. (2013). Surface engineering of inorganic nanoparticles for imaging and therapy. Adv. Drug Deliv. Rev..

[56] Xiao K., Li Y., Luo J., Lee J.S., Xiao W., Gonik A.M., Agarwal R.G., Lam K.S. (2011). The effect of surface charge on in vivo biodistribution of PEG-oligocholic acid based micellar nanoparticles. Biomaterials.

[57] Dzyhovskyi V., Romani A., Pula W., Bondi A., Ferrara F., Melloni E., Gonelli A., Pozza E., Voltan R., Sguizzato M. (2024). Characterization Methods for Nanoparticle-Skin Interaction for Nanoparticle-Skin Interactions: An Overview. Life.

[58] Robaszkiewicz A., Bartosz G., Soszyński M. (2010). N-Chloroamino acids mediate the action of hypochlorite on A549 lung cancer cells in culture. Toxicology.

[59] Kania K.D., Wagner W., Pułaski Ł. (2021). CdSe/ZnS Core-Shell-Type Quantum Dot Nanoparticles Disrupt the Cellular Homeostasis in Cellular Blood–Brain Barrier Models. Int. J. Mol. Sci..

[60] Szwed M., Wrona D., Kania K.D., Koceva-Chyla A., Marczak A. (2016). Doxorubicin–transferrin conjugate triggers pro-oxidative disorders in solid tumor cells. Toxicol. In Vitro.

[61] Sivandzade F., Bhalerao A., Cucullo L. (2019). Analysis of the Mitochondrial Membrane Potential Using the Cationic JC-1 Dye as a Sensitive Fluorescent Probe. Bio-Protocol.

[62] Szwed M., Kania K.D., Jozwiak Z. (2016). Assessment of pro-apoptotic activity of doxorubicin–transferrin conjugate in cells derived from human solid tumors. Int. J. Biochem. Cell Biol..

[63] Sadowsky C., BeGole E.A. (1981). Long-term effects of orthodontic treatment on periodontal health. Am. J. Orthod..

[64] Labieniec-Watala M., Szwed M., Hertel J., Wisnik E. (2017). Low Concentrations of Cationic PAMAM Dendrimers Affect Lymphocyte Respiration in In vitro Studies. Curr. Pharm. Biotechnol..

[65] Wigner P., Zielinski K., Labieniec-Watala M., Marczak A., Szwed M. (2021). Doxorubicin–transferrin conjugate alters mitochondrial homeostasis and energy metabolism in human breast cancer cells. Sci. Rep..

